# Clinicogenetic characterization and response to disease-modifying therapies in spinal muscular atrophy: real-world experience from a reference center in Southern Brazil

**DOI:** 10.1016/j.jped.2024.07.011

**Published:** 2024-10-16

**Authors:** Ana Letícia Amorim de Albuquerque, Júlia Kersting Chadanowicz, Isabela Possebon Bevilacqua, Ana Lucia Portella Staub, Pablo Brea Winckler, Patricia Zambone da Silva, Simone Chaves Fagondes, Renata Salatti Ferrari, Claudia Denise de Oliveira Trojahn, Viviane Zechlinski Sacharuk, Thayne Woycinck Kowalski, Karina Carvalho Donis, Michele Michelin Becker, Jonas Alex Morales Saute

**Affiliations:** a Programa de Pós-Graduação em Medicina: Ciências Médicas, Universidade Federal do Rio Grande do Sul (UFRGS), Porto Alegre, RS, Brazil; b Hospital de Clínicas de Porto Alegre (HCPA), Grupo de Pesquisa em Neurogenética Clínica, Porto Alegre, RS, Brazil; c Hospital de Clínicas de Porto Alegre (HCPA), Serviço de Neurologia, Porto Alegre, RS, Brazil; d Hospital de Clínicas de Porto Alegre (HCPA), Serviço de Fisiatria e Reabilitação, Porto Alegre, RS, Brazil; e Hospital de Clínicas de Porto Alegre (HCPA), Serviço de Pneumologia, Porto Alegre, RS, Brazil; f Hospital de Clínicas de Porto Alegre (HCPA), Serviço de Fisioterapia, Porto Alegre, RS, Brazil; g Hospital de Clínicas de Porto Alegre (HCPA), Serviço de Genética Médica, Porto Alegre, RS, Brazil; h Hospital de Clínicas de Porto Alegre (HCPA), Unidade de Neurologia Pediátrica, Porto Alegre, RS, Brazil; i Departamento de Medicina Interna, Universidade Federal do Rio Grande do Sul (UFRGS), Porto Alegre, RS, Brazil

**Keywords:** Spinal muscular atrophy, Nusinersen, Onasemnogene abeparvovec, Risdiplam, Southern Brazil

## Abstract

**Objective:**

Spinal Muscular Atrophy linked to chromosome 5q (SMA) is an autosomal recessive neurodegenerative disease characterized by progressive proximal muscle atrophy and weakness. This study addresses the scarcity of research on novel disease-modifying therapies for SMA in Latin America by reporting a real-world experience in Southern Brazil.

**Methodology:**

This is a single-center historical cohort that included all patients diagnosed with spinal muscular atrophy at a Regional Reference Service for rare diseases.

**Results:**

Eighty-one patients were included, of whom 7 died during follow-up. Of the remaining 74 patients, 5.4 % were classified as pre-symptomatic, 24.3 % with SMA type 1, 28.4 % with type 2, 36.5 % with type 3, and 5.4 % with type 4. The mean follow-up time ranged from 1.8 years for pre-symptomatic cases to 8.7 years for SMA types 2 and 3. Approximately 42 % of these patients received specific disease-modifying therapy, of these, 96.8 % received Nusinersen, with 19.4 % transitioning to gene therapy using Onasemnogene Abeparvovec, and 6.4 % starting Risdiplam. Most patients with SMA type 1 were on disease-modifying treatment, whereas only slightly over a third of patients with type 2 and about 10 % of type 3 were receiving such treatments. Among treated patients, 80 % demonstrated improvement in motor performance during the follow-up, with a lesser therapeutic response being associated with late initiation of treatment and low motor function scores at baseline.

**Conclusion:**

This real-world study reinforces the effectiveness of disease-modifying therapies for SMA in Brazil within the context of low- and middle-income countries, which is greater the earlier and the better the patient's functional status.

## Introduction

Spinal Muscular Atrophy linked to chromosome 5q is an autosomal recessive neurodegenerative disease characterized by the degeneration of motor neurons in the anterior horn of the spinal cord, resulting in progressive proximal muscle atrophy and weakness.^1^, ^2^, ^3^ Its estimated incidence is 1–1.6 in 10,000 live births, with an estimated carrier prevalence of 1 in 40 to 1 in 60 individuals in the general population.^4^^,^^5^ It is considered the leading hereditary cause of infantile death and the second most common fatal autosomal recessive disease.^1^, ^2^, ^3^

Spinal Muscular Atrophy 5q, which will be referred to in the text simply as SMA for historical reasons related to the use of the acronym, is caused by pathogenic variants in the survival motor neuron 1 (*SMN1*) gene, resulting in a marked reduction of SMN protein production, which is essential for the proper function of spinal motor neurons. In humans, in addition to the *SMN1* gene, there is a paralogous gene, *SMN2*, which differs from the former fundamentally by a single nucleotide (840C>T) in exon 7, resulting in the formation of an unstable, minimally functional SMN protein that is rapidly degraded.^1^, ^2^, ^3^ Patients with SMA-5q, due to the non-functionality of the *SMN1* gene, become dependent on the *SMN2* gene, which produces only partially functional SMN proteins, insufficient for the proper functioning of motor neurons. The disease is divided into 5 phenotypes (SMA-5q 0, I, II, III, IV) according to the age of symptom onset and the motor milestones achieved.^2^ The quantity of *SMN2* copies is the main genetic determinant of the phenotypes, with an inverse relationship between the number of *SMN2* copies and the severity of the disease.^1^, ^2^, ^3^

In recent years, a better understanding of SMA's pathophysiology has led to extraordinary advances with the confirmation of the efficacy of disease-modifying therapies. Currently, three therapies have been developed and approved by various regulatory agencies worldwide, Nusinersen,^6^ Onasemnogene abeparvovec,^7^ and Risdiplam.^8^ Nusinersen was approved by the Brazilian Health Regulatory Agency (ANVISA) in August 2017, becoming the first disease-modifying drug for SMA included in the Brazilian Unified Health System (SUS) in October 2019.^9^ In August 2020, Onasemnogene abeparvovec, and in October 2020, Risdiplam were approved by ANVISA, but it was only in May 2023 that Risdiplam was included in the national protocol for a population similar to that of Nusinersen,^10^ and in September 2023, the initial version of the inclusion of Onasemnogene abeparvovec in the protocol was published.^11^ Restrictions related to age, number of *SMN2* gene copies, and functional status, as well as the exclusion of late-onset forms such as SMA types 3 and 4, contributed to the Brazilian experience in treating SMA with disease-modifying therapies distinguishing it from that of many other countries.

Due to the scarcity of studies evaluating the experience with novel disease-modifying therapies for SMA in Latin America,^12^ this work aims to report the real-world experience of a regional Reference Service in rare diseases in Southern Brazil, following both treated patients and patients who have not yet started disease-modifying treatments.

## Methods

### Study design, population and sample

This study is a single-center historical cohort that included all patients diagnosed with spinal muscular atrophy linked to the medical genetics, neurology, and pediatric neurology divisions of the Hospital de Clínicas de Porto Alegre (HCPA) from the review of patient records between January 2012 and January 2024. The HCPA is a university hospital with 860 beds and 142 consultation rooms. Its medical genetics division is currently the only reference center for rare diseases in the state of Rio Grande do Sul, the southernmost state of Brazil, with a population of approximately 11.3 million inhabitants and about 120,000 births per year.^13^ Additionally, data from patients who underwent genetic diagnosis of SMA in the molecular genetics laboratory of the medical genetics service of HCPA and who were treated at the institution in the last two decades, from January 2000 to January 2024, were included, and their data were accounted for in the analysis. Data obtained from medical records include the disease's clinical history, SMA genetic diagnosis results, pharmacological and non-pharmacological therapies in use, and scores on clinical outcome assessments (COA).

### Clinical outcome assessments (COA)

The Children's Hospital Of Philadelphia Infant Test of Neuromuscular Disorders (CHOP INTEND, 0–64 points)^14^ and Hammersmith Infant Neurological Examination (HINE, 0–26 points) were usually applied for early-onset SMA (type 1), and the Hammersmith Functional Motor Scale Expanded (HFMSE, 0–66 points) was used for SMA types 2 and 3. In some patients with late-onset forms of SMA who were in the non-sitter or severe sitter stages, the CHOP INTEND and HINE were also applied. Since the study was a historical cohort and the data from the clinical outcome assessments (COAs) were available only for patients undergoing treatment with novel disease-modifying therapies, the scale values were presented only in the context of treatment response evaluation, with missing data reported as appropriate. Clinically relevant improvements were considered as an increase of at least 3 points on the CHOP INTEND or at least 1 point on the HINE^15^ for early-onset SMA (type 1), or at least 3 points on the HFMSE scale for SMA types 2 and 3.^16^

### Statistical analysis

Data were stored in Microsoft Office Excel and analyzed using IBM SPSS Statistics 20 for descriptive statistical analysis according to the characteristics of each variable (continuous or categorical) in mean/median and standard deviation/interquartile range or frequencies and percentages. Statistical tests were selected according to the distribution of data given by Shapiro-Wilk test and histograms.

### Ethical considerations

The study was approved by the Research Ethics Committee (CEP) of Hospital de Clínicas de Porto Alegre (CEP: CAAE No 41,550,720,900,005,327; AGHUse Research: 2020–0731). All researchers signed the Data Use Commitment Form.

## Results

The authors included 81 patients with a confirmed diagnosis of spinal muscular atrophy, whose clinical-genetic data can be seen in Table 1. Of these, 7 died during follow-up, all patients with early-onset SMA (type 1), whose causes were: 28.6 % (2) due to respiratory failure secondary to underlying disease, 28.6 % (2) due to respiratory failure secondary to pulmonary sepsis, and 42.8 % (3) due to unknown causes (no records in the medical records of the causal factor). It is noteworthy that the first molecular diagnosis made by the service was in 2001, and since then, almost every year, confirmatory genetic diagnoses have been made, with 2017 and 2018 being the years with the highest number of new diagnoses, 11 and 13 respectively. Of the 4 pre-symptomatic diagnoses, 2 were made due to a family history of an affected sibling, and 2 were made through neonatal screening in a pilot program conducted in Rio Grande do Sul.^5^

**Table 1:** Characterization of the study population.

	Pre-Symptomatic (*n* = 4)	SMA Type 1 (*n* = 25)	SMA Type 2 (*n* = 21)	SMA Type 3 (*n* = 27)^d^	SMA Type 4 (*n* = 4)
Age in months (SD)^a^	14.3 (7.2)	31.5 (20–134.5)^b^	214 (133.7)	413.3 (224.2)	423 (204.9)
Male sex % (n)	50 % (2)	48 % (12)	52 % (11)	70 % (19)	50 % (2)
Year of Diagnosis^b^	2022 (2022–2022)	2018 (2016–2019)	2017 (2014–2019)	2017 (2012–2018)	2021 (2016.5–2023)
Year of Birth n (%)					
2020–2024	4 (100 %)	4 (16 %)	0 (0 %)	0 (0 %)	0 (0 %)
2016–2019	0 (0 %)	12 (48 %)	3 (14.3 %)	0 (0 %)	0 (0 %)
2012–2015	0 (0 %)	5 (20 %)	3 (14.3 %)	3 (11.1 %)	0 (0 %)
Before 2012	0 (0 %)	4 (16 %)	15 (71.4 %)	24 (88.9 %)	4 (100 %)
Mean follow-up in years	1.8	4.8	8.7	8.7	5.5
Number of *SMN2* copies^c^ 2 copies	2 (50 %)	17 (85 %)	6 (30 %)	6 (31.6 %)	0
3 copies	0	3 (15 %)	12 (60 %)	9 (47.4 %)	2 (66.7 %)
4 copies	2 (50 %)	0	2 (10 %)	4 (21 %)	1 (33.3 %)

a Mean.

b Median (quartile 25,75).

c The number of copies of the *SMN2* gene was available for the 4 pre-symptomatic subjects, 20 for types 1 and 2 each, 19 for type 3, and 3 for type 4.

d 2 SMA type 3 patients were compound heterozygotes for the common *SMN1* deletion and for the SMN1:c.460C>T/p.(Gln154*) variant, one with 2 copies and the other with 3 copies of *SMN2*. SD, standard deviation.

Of the 74 patients currently under follow-up, 41.9 % (31) received specific therapy for SMA. Of these, 96.8 % (30) received Nusinersen, with 19.4 %^6^ of them switching to gene therapy with Onasemnogene Abeparvovec and 3.2 % (1) switching to Risdiplam. Additionally, 3.2 % (1) received Risdiplam as their first therapy. All participants who received gene therapy had SMA type 1 or were pre-symptomatic. In addition to the 31 patients currently undergoing treatment, the authors had one patient who received Nusinersen and then gene therapy but died due to respiratory complications. Of the 32 treated or undergoing treatment patients, 18 have SMA type 1 (56.25 %), 8 (25 %) type 2, 3 (9.3 %) SMA type 3, and 3 (9.3 %) are pre-symptomatic. The only pre-symptomatic patient not yet treated has 4 copies of *SMN2*.

Table 2 presents data on severity and motor and respiratory rehabilitation care, as well as the use of disease-modifying therapies, for the 74 patients currently under follow-up in the service.

**Table 2:** Clinical characteristics of SMA patients.

	Pre-Symptomatic (*n* = 4)	SMA Type 1 (*n* = 18)	SMA Type 2 (*n* = 21)	SMA Type 3I (*n* = 27)	SMA Type 4 (*n* = 4)
Non-Invasive Ventilation	0	9 (50 %)	2 (9.5 %)	3 (11.1 %)	0
Invasive Ventilation	0	8 (44.4 %)	1 (4.8 %)	0	0
Motor Physical Therapy	2 (50 %)	18 (100 %)	20 (95.2 %)	12/24 (50 %)	2 (50 %)
Respiratory Physical Therapy	0	17 (94.4 %)	9 (42.9 %)	5/25 (20 %)	0
Scoliosis	0/2 (0 %)	13/16 (81.2 %)	19/20 (95 %)	9/20 (45 %)	No data
Wheelchair Bound	0	18 (100 %)	21 (100 %)	15 (55.5 %)	2 (100 %)
Specific Therapies	3 (75 %)	17^a^ (94.4 %)	8 (38 %)	3 (11.1 %)	0
Nusinersen	3 (75 %)	16^a^ (88.9 %)	8 (38 %)	3 (11.1 %)	0
Onasemnogene abeparvovec	1 (25 %)	6^a^ (33.3 %)	0	0	0
Risdiplam	0	2 (11.1)	0	0	0

Data are presented as frequencies (percentages). Denominators were provided for all variables with missing data.

a One of the patients undergoing treatment (had received Nusinersen and switched to gene therapy with Onasemnogene Abeparvovec) died during follow-up and was not included in this table.

**Figure 1:**
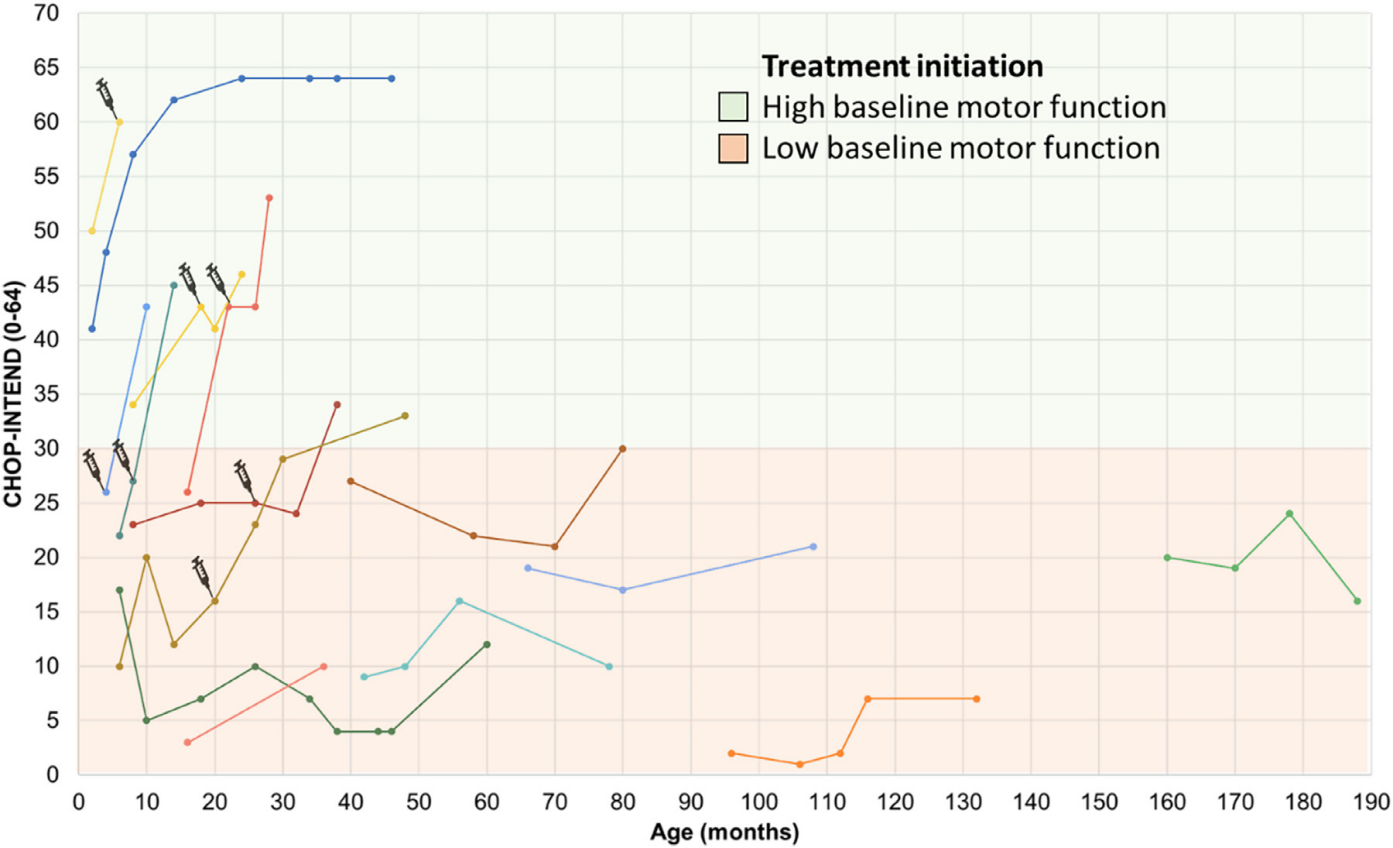
Motor performance through the CHOP INTEND scale in SMA type 1 patients undergoing treatment. The first point of each line represents the initial score on the CHOP-INTEND scale at the beginning of treatment with Nusinersen, with each line of different color representing a distinct subject. The syringe symbol represents the initiation of therapy with Onasemnogene abeparvovec. High baseline motor function, was defined as CHOP-Intend scores above 30 points according to Mendell et al.^24^ Patients undergoing treatment with Risdiplam have not yet had longitudinal follow-up and therefore do not appear in the figure. CHOP INTEND, Children's Hospital Of Philadelphia Infant Test of Neuromuscular Disorders.

**Figure 2:**
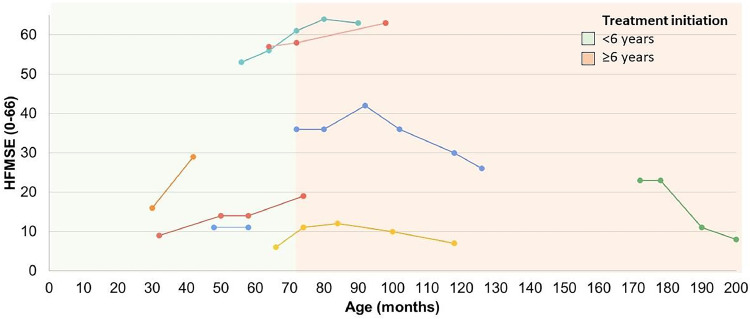
Motor performance through the Hammersmith Functional Motor Scale-Expanded (HFMSE) in SMA type 2 and type 3 patients undergoing treatment. The first point of each line represents the initial score on the HFMSE scale at the beginning of treatment with Nusinersen, with each line of a different color representing a distinct subject. The cutoff point of 6 years was established based on the stratification groups of pivotal studies of disease-modifying drugs, which considered the disease to be in an earlier or later stage based on the age at the start of treatment.^16^ HFMSE, Hammersmith Functional Motor Scale Expanded.

Figure 1, Figure 2 present the evolution in COAs of 25/32 patients treated with disease-modifying therapies on the CHOP INTEND and HFMSE. Seven patients had missing data because they did not undergo COAs.

The mean CHOP INTEND score for patients with SMA type 1 who had follow-up data (*N* = 14) at the start of treatment with disease-modifying drugs was 20 (±11.2) points (median = 21 points), increasing to 30.3 (±18.1) points (median = 31.5 points) after a mean follow-up period of 28.7 (±14.9) months, representing a mean difference of 10.2 points (95 % CI 4.0 to 16.4, *p* = 0.003, paired T-test). The maximum improvement on this scale within this group was 27 points after 12 months of follow-up, observed in a patient with SMA type 1C who had a baseline score of 26 points and started treatment at 16 months of age. The worst response (a decrease of 5 points) was seen in a patient with an initial score of 17 points who began treatment at 6 months of age (Figure 1).

The mean HFMSE score for patients with SMA types 2 and 3 who had follow-up data (*N* = 8) at the start of treatment with disease-modifying drugs was 27 (±19.5) points (median = 19.5 points), increasing to 28.3 (±22.9) points (median = 22.5 points) after a mean follow-up period of 31.6 (±15.6) months. The mean and median changes in the score were 1.2 (±9.6) and 3 points, respectively (*Z* = −0.423, *p* = 0.672, Wilcoxon test). The maximum improvement on this scale within this group was 13 points after 12 months of follow-up, observed in a patient with SMA type 2 who had a baseline score of 16 points and started treatment at 30 months of age. The worst response (a decrease of 15 points) was seen in a patient with an initial score of 23 points who began treatment at 14 years and 4 months of age (Figure 2).

In the pre-symptomatic group, all three patients (one treated at 19 days and the other two at 2 months of age) showed improvements in CHOP-Intend scores. Two of them achieved the maximum scores on the scale, and one reached a score of 60 points at 8 months of age (with a follow-up of 6 months of treatment).

When considering only clinically relevant changes in the scales, 80 % (20/25) of patients achieved them. Of these, 3 were pre-symptomatic children, 13 had SMA type 1, and the remaining 4 were part of the late-onset SMA groups (types 2 and 3). One pre-symptomatic child and 6 SMA type 1 patients previously treated with Nusinersen switched to Onasemnogene abeparvovec, with the start of use being highlighted in Figure 1. The reason for the switch was the family's preference for gene therapy due to its single-infusion treatment and their limited time to wait for the transition, considering that according to the Brazilian label, patients could only receive Onasemnogene abeparvovec up to 2 years of age. The switch was not motivated by a lack of nusinersen efficacy. The only switch from Nusinersen to Risdiplam was not due to a lack of efficacy but rather due to the increasing difficulty of intrathecal infusions caused by the progression of scoliosis.

Of note, as there was no systematic and standardized collection of information on adverse events with the new disease-modifying therapies, this information was not assessed in the records.

## Discussion

The clinical and genetic characteristics of the 74 patients with SMA from Southern Brazil are very similar to those described in the literature,^17^, ^18^, ^19^ except for finding a higher relative frequency of SMA type 3 patients with 2 copies of the *SMN2* gene. Most of the SMA type 1 patients were receiving disease-modifying treatment, whereas only just over a third of SMA type 2 patients and around 10 % of type 3 patients were also receiving it. Most patients undergoing treatment showed clinically relevant improvements in COAs, including pre-symptomatic children and patients with types 1, 2, and 3, with a lower therapeutic response associated with late treatment initiation and low baseline motor function scores.

SMA type 3 was the most frequent subtype in Southern Brazil, accounting for 33.3 % of cases, similar to what was observed in a recent Brazilian study, in which among the 450 cases evaluated, SMA type 3 was the most prevalent subtype with 40.7 % of cases, followed by SMA type 2 with 34 % and SMA type 1 with 23.3 % of cases.^19^ In the presentstudy, a significant proportion of SMA type 2 and 3 patients began follow-up many years before the data collection start date (January 2000), while a mortality rate of 28 % was observed among SMA type 1 patients during the study follow-up. Thus, it is likely that the longer survival of patients with late-onset forms influenced the results. The authors found a high frequency of SMA type 3 patients with 2 copies of *SMN2*, 30 % compared to the global frequency of 5 %.^16^ Two cases of SMA type 3 were compound heterozygotes for the common *SMN1* deletion and the SMN1:c.460C>T/p.(Gln154*) variant, which is associated with later-onset forms, regardless of the number of *SMN2* copies, one of them carrying 2 copies and the other 3 copies of *SMN2*. However, even when considering only homozygotes for the common *SMN1* deletion with SMA type 3, the frequency of subjects with 2 copies remained high (29.4 %, 5/17). The small sample size may suggest that this finding is occasional, but it is not possible to rule out that modifier genes with protective action on the phenotype may be more frequent in the studied population. Since *SMN1/SMN2* sequencing was performed only for the two compound heterozygous cases, the authors do not know if there is an increased frequency of the c.859G>C polymorphism in *SMN2*, known to attenuate the phenotype in the population, or if other intragenic variants or other modifier genes are operating to justify this result.

The authors found a higher frequency of new diagnoses in the years 2017 and 2018. However, as in 2017, there was a change in the diagnostic method of the service to MLPA (Multiplex Ligation-dependent Probe Amplification), which additionally quantified the number of *SMN2* copies, there is likely to have been an increased demand for diagnosis or prognostic information with possible therapeutic implications that would justify the increase in diagnoses in those years, not representing a real increase in incidence of cases.

Regarding non-pharmacological therapies, motor and respiratory physiotherapy are considered the standard of care for SMA.^2^, ^3^ Almost all families of SMA type 1 patients reported receiving both types of therapy, almost all SMA type 2 patients received motor physiotherapy, but just under half received respiratory physiotherapy. Half of SMA type 3 patients received motor physiotherapy and only 20 % received respiratory physiotherapy. These data suggest heterogeneity in access to different modalities of physiotherapy according to the severity of the clinical condition, with motor physiotherapy being more frequent than respiratory physiotherapy in late-onset forms. A greater frequency of physical therapy (PT) services was associated with younger age and inability to walk in previous studies in the United States, in which 85 % of SMA patients reported receiving any PT services.^20^ Additionally, the prevalence of scoliosis in the present sample for symptomatic patients varied between 45 % and 95 % depending on the groups, being slightly higher than described in the literature, with an estimated incidence between 50 % and 70 %. Further studies are needed to detail the weekly frequency and types of rehabilitation therapy to provide a more detailed understanding of access to these services by Brazilian patients, as well as their association with the development and progression of scoliosis. Such studies are essential to guide public policies that can improve the care of SMA patients in Brazil and countries with similar sociodemographic characteristics.

About 42 % of SMA patients undergoing follow-up at the reference center in Southern Brazil received at least one disease-modifying therapy for SMA. In the case of SMA type 1, 94 % (17/18) of patients were using disease-modifying therapies, demonstrating the wide availability of access to such treatments in the Brazilian Unified Health System (SUS). The only SMA type 1 patient without access to this therapy had severe previous brain injury due to cardiorespiratory complications, meeting the exclusion criteria according to the national protocol. Among SMA type 2 patients, only 38 % had access to the same type of treatment, all of whom were under 18 years of age and 75 % of them were under 10 years of age, reflecting the very recent inclusion of treatment for SMA type 2 in the SUS and some of the restrictions in the eligibility criteria of the protocol. Eighty percent of treated patients showed clinically relevant improvement in at least one of the COAs, with treatment initiation up to 18 months of age associated with relevant improvements for patients with early forms according to individual trajectories (Figure 1) and as in several other studies^2^^,^^15^^,^^21^^,^^22^^,^^23^ the earlier treated and the higher baseline motor function, characterized by CHOP-Intend scores above 30 points,^24^ the better the therapeutic responses. For late-onset forms, there were no statistically significant changes in the mean HFMSE score with treatment. However, according to individual trajectories, 83.3 % of patients who initiated treatment below 6 years of age (the stratification cutoff point for randomization in pivotal clinical trials with disease-modifying therapies for SMA)[16] showed clinically relevant improvement during the course of treatment (not sustained in one of them). Only two patients with available data had started treatment at 6 years of age or older, one of them showed clinically relevant but not sustained improvement and the other showed worsening of motor scores (Figure 2). It is worth noting that different studies have shown improvement in motor scores with disease-modifying treatments in late-onset forms, especially in SMA type 2 patients, but also in type 3, something that is not observed in the natural history of the disease.^12^^,^^16^

Three out of four pre-symptomatic SMA children were receiving disease-modifying treatments, all of them had motor scores close to normal for their age range. Different studies with disease-modifying therapies for SMA showed very similar motor performance to that of healthy children when treatment is initiated early in the pre-symptomatic phase.^22^^,^^23^ These data highlight the importance of neonatal screening, which has already been carried out in North America, Europe, and Asia,^23^ and is being pioneered in Brazil.^5^

All patients undergoing gene therapy who underwent more than two COA assessments had clinically significant responses, with mean increases of 109 % for CHOP-INTEND (Figure 1) and 166.5 % for HINE. However, it is not possible to assert that such patients had clearly additional benefits to what they were already achieving with Nusinersen treatment. One of the patients who received gene therapy died in the first month post-treatment due to ventilatory failure, considered an adverse event unrelated to the medication.

Access to disease-modifying therapies for SMA patients in Brazil began in 2019 when Nusinersen was included in the Brazilian Unified Health System through a clinical protocol and therapeutic guideline.^9^ The initial protocol was restricted to SMA type 1 patients who were not dependent on mechanical ventilation 24 h a day and for pre-symptomatic use by children with up to 3 copies of *SMN2*. Access to therapies for SMA type 2 patients in the Brazilian protocol only occurred in November 2022, when the national clinical protocol for SMA expanded the use of Nusinersen to include SMA type 2 patients up to 12 years of age at the start of treatment or who had preserved the ability to sit without support as well as upper limb function.^25^ Prior to the publication of the national protocol, there were expert notes from different Brazilian medical societies suggesting that it was not possible to wait for the elaboration of a protocol to initiate treatment in SMA type 2 patients under 5 years of age or who presented rapid progression of the disease.^26^ The higher frequency of SMA type 2 patients under 10 years of age in treatment in the present study is probably related to this conduct and to the probable access of families through litigation. Additionally, many SMA type 2 patients over 10 years of age had severe scoliosis or had already undergone spinal arthrodesis, which made it difficult or even contraindicated intrathecal infusion of Nusinersen. However, it is worth noting that the frequency of patients with SMA type 2 treated in other centers in Brazil may vary, with positive experiences reported from other centers regarding the administration of nusinersen, even in cases with previous spinal surgery.^27^ More recently, in May 2023, Risdiplam was included in the national protocol for a population similar to that of Nusinersen,^10^ which, being administered orally, has no restrictions related to the presence of severe scoliosis in treatment eligibility, and it is likely that there will be a significant increase in the proportion of SMA type 2 patients undergoing treatment in the coming years. In September 2023, the initial version of the inclusion of Onasemnogene abeparvovec in the protocol was published,^11^ restricted to SMA type 1 patients up to 6 months of age. Additionally, Article 196 of the Brazilian Federal Constitution, considering that health is a right of all and a duty of the state, ends up being the starting point for the judicialization of access to high-cost treatments.^28^ Through punctual interventions by the judiciary, many SMA patients were treated outside the incorporation criteria mentioned above, resulting in greater heterogeneity in access to these treatments, which in part is reflected in the results presented in the study.

### Study limitations

The fact that it is a single-center study may limit the external validity of the data, however, it increases the homogeneity in data collection and thereby the robustness of the information. Although scoring on COAs specific to SMA during follow-up of patients using disease-modifying therapies was a prerequisite for the Brazilian protocol, since the study was retrospective and since a large part of the data was recorded during the COVID-19 pandemic, longitudinal COA data were not available for 21.8 % of patients undergoing treatment. This rate of missing data related to longitudinal follow-up of COAs is also due to cases with access to treatments through litigation, where the scales were applied externally to the reference centers and sometimes there was no record of the score or they were simply not performed because there was no obligation to do so. Another limitation is related to potentially undiagnosed cases in Rio Grande do Sul or cases that have not been referred to the rare disease reference center, particularly cases where the symptoms are mild and may not have been diagnosed yet, and cases of type 0 and type 1, which have severe symptoms and die before diagnosis.

## Conclusion

In conclusion, this real-world study reinforces the efficacy of disease-modifying therapies for SMA in Brazil and in the context of countries with similar socioeconomic profiles, which is greater the earlier and the better the patient's functional status. Furthermore, the great heterogeneity of access to such treatments among the different types of SMA, used in almost all patients with early forms and in a minority of SMA type 3 patients, reinforces the importance of national protocols in the context of the Brazilian SUS for effective access to such therapies. Even with the possibility of judicialization of access, this ends up being infrequent when patients do not meet the eligibility criteria of the protocols. In addition, the study points to the need for greater detail on access and type of rehabilitation treatments that are performed in the Brazilian population.

## Conflicts of interest

AMS is the principal investigator in clinical trials conducted by both Biogen and Novartis, companies that have commercial treatments for spinal muscular atrophy. The other authors declare no conflicts of interest to disclose.
